# Roles of bone-derived hormones in type 2 diabetes and cardiovascular pathophysiology

**DOI:** 10.1016/j.molmet.2020.101040

**Published:** 2020-06-13

**Authors:** Xuzhu Lin, Danise-Ann Onda, Chieh-Hsin Yang, Joshua R. Lewis, Itamar Levinger, Kim Loh

**Affiliations:** 1 St. Vincent's Institute of Medical Research, Fitzroy, VIC, Australia; 2 Department of Medicine, University of Melbourne, Parkville, VIC, Australia; 3 School of Medical and Health Sciences, Edith Cowan University, Perth, Australia; 4 Medical School, University of Western Australia, Perth, Australia; 5 Institute for Health and Sport (IHES), Victoria University, Footscray, VIC, Australia; 6 Australian Institute for Musculoskeletal Science (AIMSS), University of Melbourne and Western Health, St Albans, VIC, Australia

**Keywords:** Bone-derived hormones, Osteocalcin, Lipocalin 2, Sclerostin, Cardiometabolic health, Type 2 diabetes, Cardiovascular disease, Chronic inflammation

## Abstract

**Background:**

Emerging evidence demonstrates that bone is an endocrine organ capable of influencing multiple physiological and pathological processes through the secretion of hormones. Recent research suggests complex crosstalk between the bone and other metabolic and cardiovascular tissues. It was uncovered that three of these bone-derived hormones—osteocalcin, lipocalin 2, and sclerostin—are involved in the endocrine regulations of cardiometabolic health and play vital roles in the pathophysiological process of developing cardiometabolic syndromes such as type 2 diabetes and cardiovascular disease. Chronic low-grade inflammation is one of the hallmarks of cardiometabolic diseases and a major contributor to disease progression. Novel evidence also implicates important roles of bone-derived hormones in the regulation of chronic inflammation.

**Scope of review:**

In this review, we provide a detailed overview of the physiological and pathological roles of osteocalcin, lipocalin 2, and sclerostin in cardiometabolic health regulation and disease development, with a focus on the modulation of chronic inflammation.

**Major conclusions:**

Evidence supports that osteocalcin has a protective role in cardiometabolic health, and an increase of lipocalin 2 contributes to the development of cardiometabolic diseases partly via pro-inflammatory effects. The roles of sclerostin appear to be complicated: It exerts pro-adiposity and pro-insulin resistance effects in type 2 diabetes and has an anti-calcification effect during cardiovascular disease. A better understanding of the actions of these bone-derived hormones in the pathophysiology of cardiometabolic diseases will provide crucial insights to help further research develop new therapeutic strategies to treat these diseases.

## List of abbreviations

8-iso-PGF2α: 8-iso-prostaglandin F2α
AP-1: activator protein 1
ApoE: apolipoprotein E
ATF6: activating transcription factor 6
BAT: brown adipose tissue
cOC: carboxylated OC
CVD: cardiovascular disease
CXCR2: the chemokine C-X-C motif receptor 2
eNOS: endothelial nitric oxide synthase
ER: endoplasmic reticulum
ERK1/2: extracellular signal-regulated protein kinase 1/2
FAs: fatty acids
GLP-1: glucagon-like peptide-1
Glu: glutamine
GPRC6A: the G protein-coupled receptor family C group 6 member A
GPx-1: glutathione peroxidase 1
GSH: glutathione
GSSG: glutathione disulfide
HAECs: human aortic endothelial cells
HASMCs: human aortic smooth muscle cells
HFD: high-fat diet
HPASMCs: human pulmonary artery smooth muscle cells
hs-CRP: high sensitivity C-reactive protein
HUVECs: human umbilical vein endothelial cells
IKK: the IκB kinase
IL-6: interleukin-6
IRE-1: inositol-requiring kinase 1
JNK: c-Jun N-terminal kinase
KDa: kilo-Dalton
LCN2: lipocalin 2
LDL: low-density lipoprotein
LOX-1: lectin-type oxidized LDL receptor 1
LPS: lipopolysaccharides
LRPs: LDL receptor related proteins
MC4R: melanocortin-4 receptor
MCP-1: monocyte chemoattractant 1
MDA: malondialdehyde
NASH: non-alcoholic steatohepatitis
NF-κB: the nuclear factor kappa-light-chain-enhancer of activated B cells
NGAL: neutrophil gelatinase-associated lipocalin
NLRP3: nucleotide-binding domain-like receptor protein 3
NO: nitric oxide
Nrf2: nuclear factor erythroid 2-related factor 2
OC: osteocalcin
PERK: the double-stranded RNA-activated protein kinase-like ER kinase
SOD: superoxide dismutase
SRA-1: scavenger receptor A-1
T2D: type 2 diabetes
TNF-α: tumor necrosis factor-alpha
ucOC: undercarboxylated OC
WAT: white adipose tissue

## 1 Introduction

Type 2 diabetes (T2D) and cardiovascular disease (CVD) and two of the most prevailing cardiometabolic disorders in Western society and are closely linked in terms of pathophysiology [1]. The risk of developing CVD in individuals with T2D is two-fold higher, and CVD is the major cause of mortality in individuals with T2D [2]. Furthermore, a cluster of risk factors, including inflammation, obesity, dyslipidemia, and insulin resistance appears to be unifying causal factors in both diseases [3]. Therefore, a greater understanding of pathophysiological commonalities in T2D and CVD is critical for developing new precautionary measures and treatments to simultaneously combat these diseases.

Both T2D and CVD are closely linked with bone diseases such as bone fragility and osteoporosis [4,5]. The traditional notion that bone is an inert organ primarily responsible for body protection and locomotion led to assumptions that these symptoms in bone are merely outcomes of T2D and CVD. However, in the last decade, an increasing amount of evidence has proposed that bone as an endocrine organ that influences various organs and tissues via the secretion of multiple bone-derived hormones [6]. These endocrinal functions are, for example, glucose homeostasis [7], appetite control [8], fat deposition [9], skeletal muscle adaptation [10], male fertility [11], and cognition [12]. Many comprehensive studies have shown that three of these hormones—osteocalcin (OC), lipocalin 2 (LCN2), and sclerostin—are the most closely linked to energy metabolism and cardiovascular health [[7], [8], [9],[13], [14], [15]]. This brand-new concept regarding bone functions raises a possibility for dynamic crosstalk between bone and metabolic tissues/vasculature during the development of T2D and CVD. In this review, we will examine the evidence for the roles of OC, LCN2, and sclerostin in metabolic and cardiovascular events. One focus is on novel assessment of potential roles and mechanisms of each hormone in the modulation of chronic inflammation.

## 2 Chronic inflammation during the development of type 2 diabetes and cardiovascular disease

Increasing evidence has suggested that chronic inflammation is a common underlying factor shared by T2D and CVD pathologies [16,17]. Compared with acute inflammation, induced by infections or autoimmune reactions, the dimensions of the chronic inflammatory state are moderate [18]. As such, the state is often referred to as “low-grade chronic inflammation” or “metabolic inflammation” [3]. Although the exact underlying mechanisms have not been fully elucidated, chronic inflammation is believed to play critical roles in various stages of T2D and CVD development. Inflammatory reactions are often generated by nutrition-excess-induced obesity, which is largely attributed to a Western-style diet. In such circumstances, adipocyte hypertrophy facilitates cell rupture [19], endoplasmic reticulum (ER) [20], and oxidative stress [21]. These conditions lead to the enhanced expression of inflammatory markers such as interleukin-6 (IL-6), tumor necrosis factor-alpha (TNF-α), IL-1β, and monocyte chemoattractant 1 (MCP-1) via activation of various inflammatory pathways, such as the nuclear factor kappa-light-chain-enhancer of activated B cells (NF-κB) and c-Jun N-terminal kinase (JNK) pathways [3]. Cell apoptosis and pro-inflammatory factor production in adipocytes subsequently attract and activate tissue-resident macrophages. These activated macrophages are the main source of inflammatory mediators that leads to exacerbations in inflammatory responses and essential for insulin resistance in adipose tissue [22]. As a consequence, enhanced levels of circulation based free fatty acids (FAs), glucose, and pro-inflammatory factors induce fat accumulation and macrophage infiltration in other organs or tissues, including the liver [23], skeletal muscle [24], and arterial walls [25]. These events promote systemic inflammatory responses, which in combination with other metabolic abnormalities such as hyperlipidemia and hyperglycemia ultimately lead to T2D and CVD development [26].

OC, LCN2, and sclerostin have been linked with inflammatory activities in pathological conditions such as bacterial infection and rheumatoid arthritis [[27], [28], [29]], before the identification of their endocrinal functions. More recent findings related to the regulations in cardiometabolic health by these bone-derived hormones have attracted considerable interest in characterizing their roles in the regulation of chronic inflammation during T2D and CVD. Evidence shows that their effects on inflammation are as follows: regulate inflammatory reactions in metabolic and cardiovascular tissues, and modulate the activity and behaviors of immune cells such as monocytes, macrophages, and neutrophils.

### 2.1 Osteocalcin overview

OC is a major non-collagenous protein in bone, predominantly produced by osteoblasts [30]. Human OC is a 5.7 kilo-Dalton (KDa) peptide containing 49 amino acids (mouse OC has 46 amino acids). During post-translational modifications, OC can be carbonylated at its 17th, 21st, and 24th glutamine (Glu) residues into carboxylated OC (cOC) [31]. In bone, there remains a small proportion of OC with one or more uncarboxylated Glu residues (termed undercarboxylated OC [ucOC]), and in the general circulation where the percentage of ucOC is much higher (40%–60%) with physiological serum levels normally below 30 ng/mL (in mice < 50 ng/mL) [32,33]. Although the paracrinal functions of OC in bone have not been fully elucidated, it has been shown that OC suppresses bone formation and mineralization, without impairing bone resorption [34,35]. Many studies have demonstrated that OC functions as a bone-derived hormone involved in multiple biological processes, including energy metabolism [7], cardiovascular health [36], male fertility [11], cognition [12], and stress response [37]. These physiological functions have been recognized to be primarily regulated by ucOC [31], although some studies have suggested that cOC may also be biologically active [38,39].

### 2.2 Beneficial roles of osteocalcin in energy metabolism and metabolic disorders

**Figure 1:**
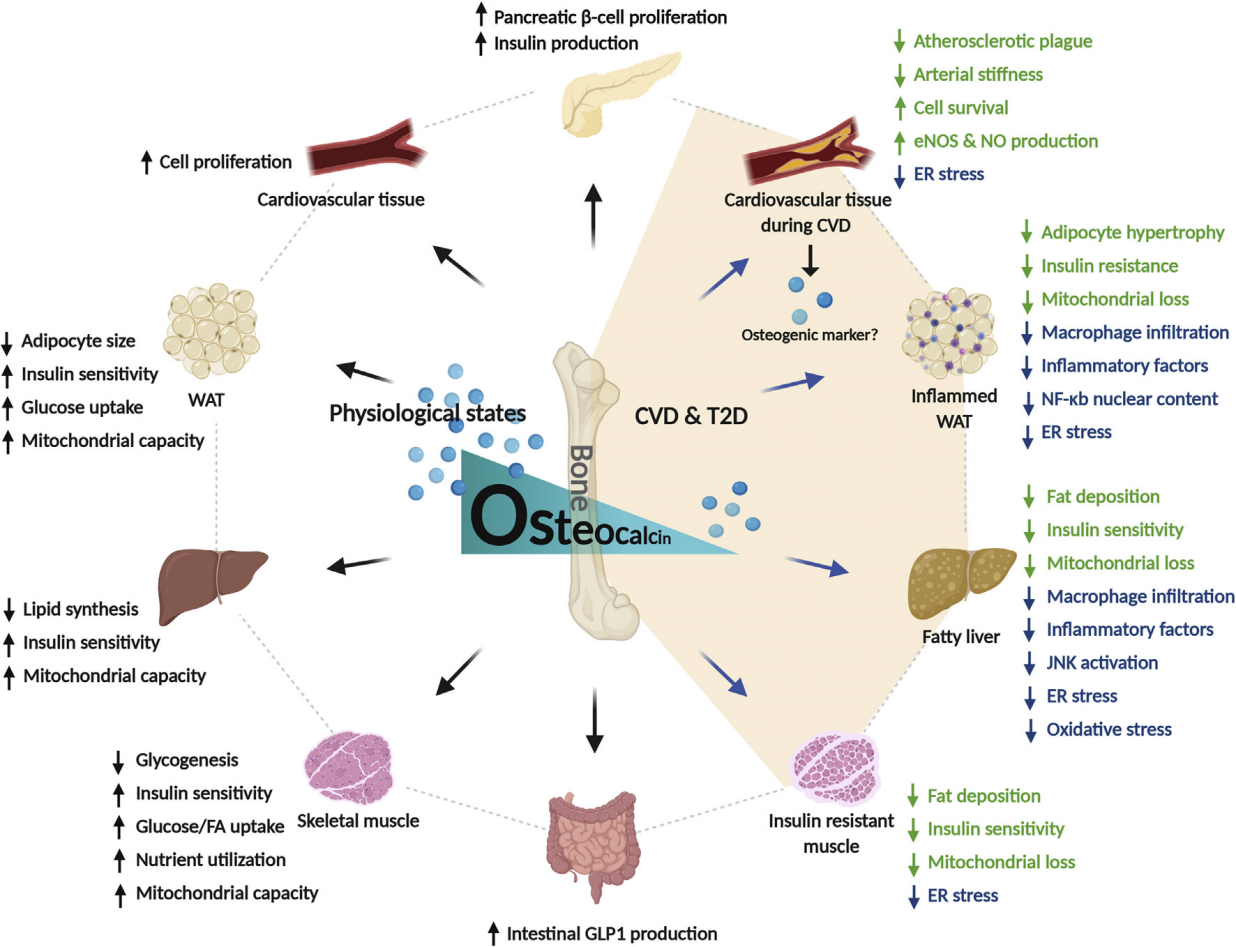
**Roles of osteocalcin in cardiometabolic regulation and disease**. Figure 1 incorporates findings from animal models and humans. The majority of OC, including its active form ucOC, is produced by osteoblasts [30]. During CVD, cardiovascular tissues also express OC to a much lesser extent, probably as an osteogenic marker [59,60]. The literature suggests that ucOC benefits energy metabolism and cardiovascular health in physiological states. First, ucOC promotes β-cell proliferation and insulin production in pancreas [42,46]. ucOC also indirectly favors insulin production via enhancing GLP-1 secretion from intestine [43]. Furthermore, in insulin target tissues such as WAT, liver, and muscle, ucOC suppresses adipocyte size [46], increases glucose and FA uptake [10,44], enhances insulin sensitivity [45], and promotes nutrient utilization and mitochondrial capacity [10]. ucOC also reduces lipid synthesis in liver and glycogen production in muscle [10,49]. ucOC also enhances cell proliferation in HAECs and HASMCs [61]. During T2D and CVD, ucOC levels are decreased [[50], [51], [52]]. In insulin target tissues, the administration of ucOC suppresses excessive fat deposition, ameliorates insulin resistance, and restores impaired mitochondrial capacity [47,86]. The administration of ucOC has also been demonstrated to exert rescuing effects on vasculature during CVD. ucOC has been demonstrated to reduce atherosclerotic plaque formation and arterial stiffness [14,58]. In various types of vascular cells, ucOC enhances cell survival and expression of eNOS and NO [14,62,63]. Numerous studies suggest an anti-inflammatory role of ucOC in cardiometabolic syndromes. In WAT and liver, ucOC reduces macrophage infiltration [78], inflammatory factor expression [39], and the activation/translocation of pro-inflammatory nuclear factors [76]. ucOC also decreases ER stress in insulin target tissues and vascular cells [14,48]. Moreover, ucOC attenuates inflammation in liver by mitigating oxidative stress [75].Short-head arrows: secretion. Long-head arrows: suggested OC effects. Green texts: direct beneficial effects on cardiometabolic tissues in T2D or CVD conditions. Blue texts: anti-inflammatory effects in T2D or CVD conditions. OC – osteocalcin; ucOC – undercarboxylated osteocalcin; T2D – type 2 diabetes; CVD – cardiovascular disease; GLP-1– glucagon-like peptide-1; WAT – white adipose tissue; FA – fatty acid; HAECs – human aortic endothelial cells; HASMCs– human aortic smooth muscle cells; eNOS – endothelial nitric oxide synthase; NO – nitric oxide; ER – endoplasmic reticulum.

A considerable amount of evidence in mice supports that ucOC improves glucose homeostasis and energy balance (Figure 1) [31,32]. In 2007, Lee et al. showed that an OC deficiency in mice led to adipocyte hypertrophy, insulin resistance, glucose intolerance, and high circulatory triglyceride levels [7]. By contrast, in the “gain-of-function” mouse model, which had higher ucOC levels, mice exhibited hypoglycemic phenotypes [7]. Moreover, mice deficient in the G protein-coupled receptor family C group 6 member A (GPRC6A), the putative receptor for ucOC, exhibited metabolic syndrome similar to that reported for OC^−/-^ mice [40]. This milestone paper not only proved that OC plays an important role in energy and glucose homeostasis but also—for the first time—showed that a hormone produced by bone regulates physiological processes not directly linked with bone maintenance. While two recent studies showed no metabolic abnormalities in mice with osteocalcin deficiency (Diegel et.al 2020 Plos Genetics & Moriishi et.al 2020 Plos Genetics), these discrepancies could be attributed to differences in genetic modification methods, mouse genetic backgrounds or housing environments [164,165]. Nonetheless, strong evidence suggested that ucOC exerted its metabolic effects by targeting multiple tissues essential for glucose and lipid metabolism. In the pancreas, ucOC has been reported to directly increase cell proliferation, *Ins* expression, and insulin secretion in β-cells [41,42]. ucOC may also enhance insulin secretion in the pancreas by increasing the production of glucagon-like peptide-1 (GLP-1) from the intestines [43]. In major insulin target tissues, such as white adipose tissue (WAT), liver, and skeletal muscle, ucOC directly enhances glucose and FA uptake [10,39,44], insulin sensitivity [[45], [46], [47]], nutrient utilization [10,39], and mitochondrial capacity [10,41,48] and reduces glycogen and lipid synthesis [10,49].

Notably, literature has reported that the circulating levels of ucOC are reduced in both humans and mice in the presence of metabolic syndromes such as insulin resistance and T2D [[50], [51], [52]] and that these disorders can be ameliorated by administering ucOC (Figure 1) [7,38,41,[46], [47], [48],^52^]. ucOC administration to obese diabetic mice has been demonstrated to improve systemic glucose tolerance and insulin sensitivity, concomitant with reductions in hyperlipidemia and whole-body adiposity [38,41,48]. Consistently, the overexpression of ucOC protected mice from obesity and glucose intolerance induced by gold thioglucose injection [7]. In insulin-resistant muscle, liver, and WAT, the treatment of ucOC restores impaired response to insulin stimulation, perturbed energy metabolism, and compromised mitochondrial capacity [[46], [47], [48],^52^].

The human evidence related to a possible causal relationship between OC-GPRC6A axis and energy metabolism appears contradictory to the findings of mice studies. Genetic evidence suggests that a loss-of-function mutation in GPRC6A was accompanied by glucose intolerance in two patients [11]. Furthermore, a polymorphism rs1800247 in the OC gene and a rs2274911 polymorphism in the GPRC6A gene have been be associated with insulin resistance [53,54]. However, two recent studies have suggested limited effects of ucOC in humans by reporting that ucOC reduction, via a 3-year Vitamin K supplementation, had no impact on body composition in older men and women [55,56]. Thus, it remains too early to conclude whether the metabolic effects of ucOC reported in mice models also exist in humans. To clarify the therapeutic potential of ucOC, further research is necessary to reveal metabolic regulations via ucOC in human tissues and cells.

### 2.3 Beneficial role of osteocalcin in protecting cardiovascular health

Unlike ucOC metabolic functions, OC mechanistic insights in cardiovascular health are fewer and equivocal. Several animal studies have suggested that ucOC may protect vasculature under T2D conditions (Figure 1) [14,57,58]. For instance, ucOC administration improved diastolic blood pressure, endothelium-dependent relaxation, and aortic atherosclerotic plaque formation in high-fat diet (HFD)-fed apolipoprotein E (ApoE)^−/−^ mice [14]. In diabetic rats, the enhancement of circulatory ucOC, either by ucOC or warfarin administration, improved arterial stiffness, vascularization, capillary density, and neovascularization and decreased myocardial fibrosis [57,58]. However, whether these improvements are from ucOC acting directly on vascular cells or are as a consequence of improved metabolic outcomes remains unknown.

Evidence from *in vitro* studies has demonstrated direct protective effects of ucOC on the vasculature [14,[59], [60], [61], [62], [63]]. The treatment of ucOC at physiological levels was reported to enhance cell proliferation in human aortic endothelial cells (HAECs) and human aortic smooth muscle cells (HASMCs) [61]. In pathological conditions, ucOC treatment also exerted protective effects on multiple cell types in human vessel walls (i.e., aortic endothelial, umbilical vein endothelial, and smooth muscle cells). These protective effects were generated by increasing the activation and expression of endothelial nitric oxide synthase (eNOS) and nitric oxide (NO) [14,62,63] and reducing cell apoptosis induced by FAs [62]. ucOC treatment (10 ng/mL) also attenuated atherogenic diet-induced impairment of endothelium-dependent vasorelaxation in the rabbit aorta [36]. Nevertheless, compared with circulating ucOC secreted from bone, locally expressed OC in vascular smooth muscle cells [59,60], endothelial progenitor cells [64], platelets [65], and monocytes [66] have been associated with arterial calcification—a hallmark of advanced atherosclerosis. However, because OC has been one of the best molecular markers for osteogenic transcriptional programs [67], whether non-bone-derived OC is an active player potentiating CVD development or merely a osteogenic marker requires further investigation.

These findings suggest direct protective effects of OC on vasculature during CVD, athough the overall effects of ucOC on cardiovascular health may be mainly from its metabolic effects.

### 2.4 Anti-inflammatory role of osteocalcin in cardiometabolic disorders

As described, ucOC probably has therapeutic effects against T2D and CVD [7,14]. Therefore, a possibility is that ucOC exerts these beneficial effects, at least partially, by suppressing chronic inflammation. Indeed, accumulating correlational evidence has shown that OC perturbations in T2D and CVD are associated with inflammation, for example, high sensitivity C-reactive protein (hs-CRP), a marker of systemic inflammation, was reported to be inversely associated with circulating total OC and ucOC levels in patients with T2D [[68], [69], [70]]. Other inflammatory markers such as TNF-α, IL-6, and ferritin were also negatively associated with serum ucOC levels in T2D or pre-diabetic patients [71,72]. In addition, ucOC was negatively correlated with several chronic inflammation parameters, including total leukocyte, neutrophil, and monocyte counts in T2D patients [73].

The anti-inflammatory roles of ucOC have been investigated in several mechanistic studies, using both *in vivo* and *in vitro* models (Figure 1) [74,75]. The administration of ucOC to ApoE^−/−^ mice challenged with a HFD reduced circulatory levels of several inflammatory cytokines, including TNF-a, IL-1a, and IL-12 [14]. In WAT, ucOC administration reportedly decreased macrophage infiltration in mice with hypothalamic obesity [76]; it also reduced the expression of macrophage markers and inflammatory genes such as *Cd68*, *F4/80*, *Mcp1*, *Tnf*, *Nlrp3*, *Ciita*, and *Cd74,* in lean [77] and HFD-fed mice [74]. In the liver, ucOC suppressed diet-induced JNK activation and inflammatory gene expression [74,75]. Notably, these anti-inflammatory ucOC effects can probably be passed to offspring via epigenetic inheritance, because male pups from ucOC-administered female mice were protected from high energy diet-induced pro-inflammatory marker expression, and macrophage infiltration in both WAT and liver [78]. In *in vitro* models, ucOC treatment (20 ng/mL) in 3T3-L1 adipocytes attenuated TNF-α-induced enhancement of several inflammatory marker genes, including *Tnf*, *Ccl2*, *Nfkb1*, and *Rela*, and the nuclear content of the NF-κB p65 subunit [76]. In primary rat adipocytes, ucOC at 20 ng/mL also suppressed TNF-α production [39].

In contrast to the aforementioned evidence, suggesting an anti-inflammatory role for ucOC, other evidence has indicated that the effects of ucOC on inflammatory gene regulation may be multifaceted. For example, ucOC does not influence IL-6, IL-8, or MCP-1 expression levels in either acute or chronic inflammation in *in vitro* human vascular cells [79]. Additionally, ucOC treatment enhanced IL-6 expression in adipocytes [39] and myotubes [10].

Collectively, these data suggest that ucOC ameliorates inflammation in mice with metabolic disorders, especially in WAT and the liver. However, because ucOC has profound metabolic effects, whether these proposed anti-inflammatory effects are merely outcomes from the reduction of hyperglycemia and hyperlipidemia remains unclear. Thus, further research is necessary to show that ucOC directly regulates inflammatory factors and signaling pathways, under pathological and metabolically inflammatory conditions. One question that must be addressed is whether ucOC exerts its effects on myeloid lineage cells, because macrophages play key roles in insulin resistance and atherosclerosis. Although there is no direct evidence, the likelihood of a direct effect of ucOC on myeloid cells is high because the expression of GPRC6A has been found in mouse and human monocytes [80] and in peritoneal [81] and RAW264.7 macrophages [82].

### 2.5 Osteocalcin-induced suppression of endoplasmic reticulum stress and oxidative stress

A potential mechanism for how ucOC directly ameliorates chronic inflammation in different cells and tissues is the suppression of ER stress (Figure 1). As aforementioned, ER stress appears to provoke inflammation in T2D and CVD. Under these conditions, the build-up of ER stress via nutrient overload [83], unfolded protein accumulation [83], and high lipid levels [84] triggers signaling pathways modulated by three ER based sensing proteins: double-stranded RNA-activated protein kinase-like ER kinase (PERK), inositol-requiring kinase 1 (IRE-1), and activating transcription factor 6 (ATF6) [85]. Notably, the activation of ER stress pathways leads to JNK and the IκB kinase (IKK) phosphorylation, which translocates nuclear factor activator protein 1 (AP-1) and NF-κB, thereby promoting inflammatory factor expression [85].

In mice, ucOC administration has been demonstrated to inhibit the HFD-induced enhancement of PERK and IRE-1α phosphorylation and ATF6β expression in WAT, liver, skeletal muscle, and aorta cells [47,48,86]. In addition, *in vitro* ucOC treatment (5 ng/mL) reduced PERK and IRE-1α phosphorylation, and ATF6β expression induced by tunicamycin, in 3T3–L1 adipocytes, Fao liver cells, L6 muscle cells, vascular endothelial cells, and vascular smooth muscle cells [47,48,86]. Notably, NF-κB activation was involved in the suppression effects of ucOC on ER stress in *in vitro* models [48]. However, whether ucOC administration suppressed the expression of pro-inflammatory factors was not reported. Moreover, because NF-κB activation is widely accepted as a central mediator in nutrient excess-induced inflammation [87], the proposed mechanisms based on *in vitro* models may not explain the ucOC effects on ER stress *in vivo*. Therefore, whether and how ucOC suppresses chronic inflammation via ER stress relief requires further exploration.

ucOC has been suggested to suppress chronic inflammation via oxidative stress mitigation (Figure 1). Oxidative stress is implicated in several molecular inflammatory events during T2D and CVD, namely, low-density lipoprotein (LDL) oxidation [88] and the activation of nucleotide-binding domain-like receptor protein 3 (NLRP3) inflammasomes [89]. In the liver of HFD-fed mice, ucOC administration reduced oxidative stress biomarker expression, including malondialdehyde (MDA), 8-iso-prostaglandin F2α (8-iso-PGF2α), and glutathione disulfide (GSSG)/glutathione (GSH) ratios [75]. This hepatic oxidative stress relief was probably due to the up-regulation of other antioxidant enzymes, including catalase, superoxide dismutase (SOD), and glutathione peroxidase 1 (GPx-1), via activation of the nuclear factor, nuclear factor erythroid 2-related factor 2 (Nrf2) [75]. In another study, ucOC suppressed oxidative stress induced by high glucose levels in MC3T3-E1 cells [90]. However, as the evidence is scarce and suggestive, further investigations are required to discern the possible effects of ucOC on oxidative stress.

### 2.6 Lipocalin 2 overview

LCN2, also termed 24p3 or neutrophil gelatinase-associated lipocalin (NGAL), is a 25 kD glycoprotein that in physiological states (normally <100 ng/mL in human serum [91], <200 ng/mL in mouse serum [8]) has been linked with numerous functions such as neutrophil function [92], mechanoresponsive gene regulating bone homeostasis in response to exercise/inactivity [93], and skeletal muscle regeneration [94]. It has been suggested that in physiological state lipocalin 2 is polyaminated [95] and expressed predominantly from bone cell osteoblasts [8]. In bone, LCN2 is regulated by physical activity/inactivity [93] and negatively modulates bone development and turnover, as LCN2 overexpression in mouse bone results in thinner cortical bone and reduces osteoblast differentiation [96]. Recently, bone-derived LCN2 was shown to have endocrinal functions in appetite control and insulin secretion in mice [8]. However, in pathological states, LCN2 expression can probably be induced in a wide range of cells and tissues, including adipose tissue [97,98], kidney [99], liver [100], and vasculature [101]. As such, increased circulating LCN2 is often used as a pathological biomarker such as acute kidney injury [102]. In pathological conditions, it was shown that two other forms of LCN2—R81E and deamidated LCN2—were expressed at least in WAT [95,103]. Thus, pathological forms of LCN2 probably display diverse ligand-binding and post-translation modifications, depending on the different tissue/cell sources, further complicating their causal roles in acute and chronic diseases.

### 2.7 Endocrine regulations of energy metabolism by lipocalin 2

Recent evidence has suggested that LCN2 plays a vital role in energy metabolism and is closely related to diabetes [8,104,105]. Human studies have demonstrated that higher circulating LCN2 levels are associated with obesity, insulin resistance, and dyslipidemia in T2D patients, thereby proposing LCN2 as a serum marker for metabolic disorders [106,107]. Some evidence from animal models has also linked adverse metabolic effects to LCN2 [[108], [109], [110]]. Law et al. showed that LCN2 deficiency protected mice from aging and obesity-induced insulin resistance [110]. In another study, mice with systemic LCN2 overexpression, exhibited enhanced fat mass and adipocyte size, accompanied by increased food intake, glucose intolerance, and insulin resistance [109]. Additionally, Jun et al. reported that LCN2^−/−^ mice exhibited modestly improved glucose tolerance compared with that of their WT littermates [108].

**Figure 2:**
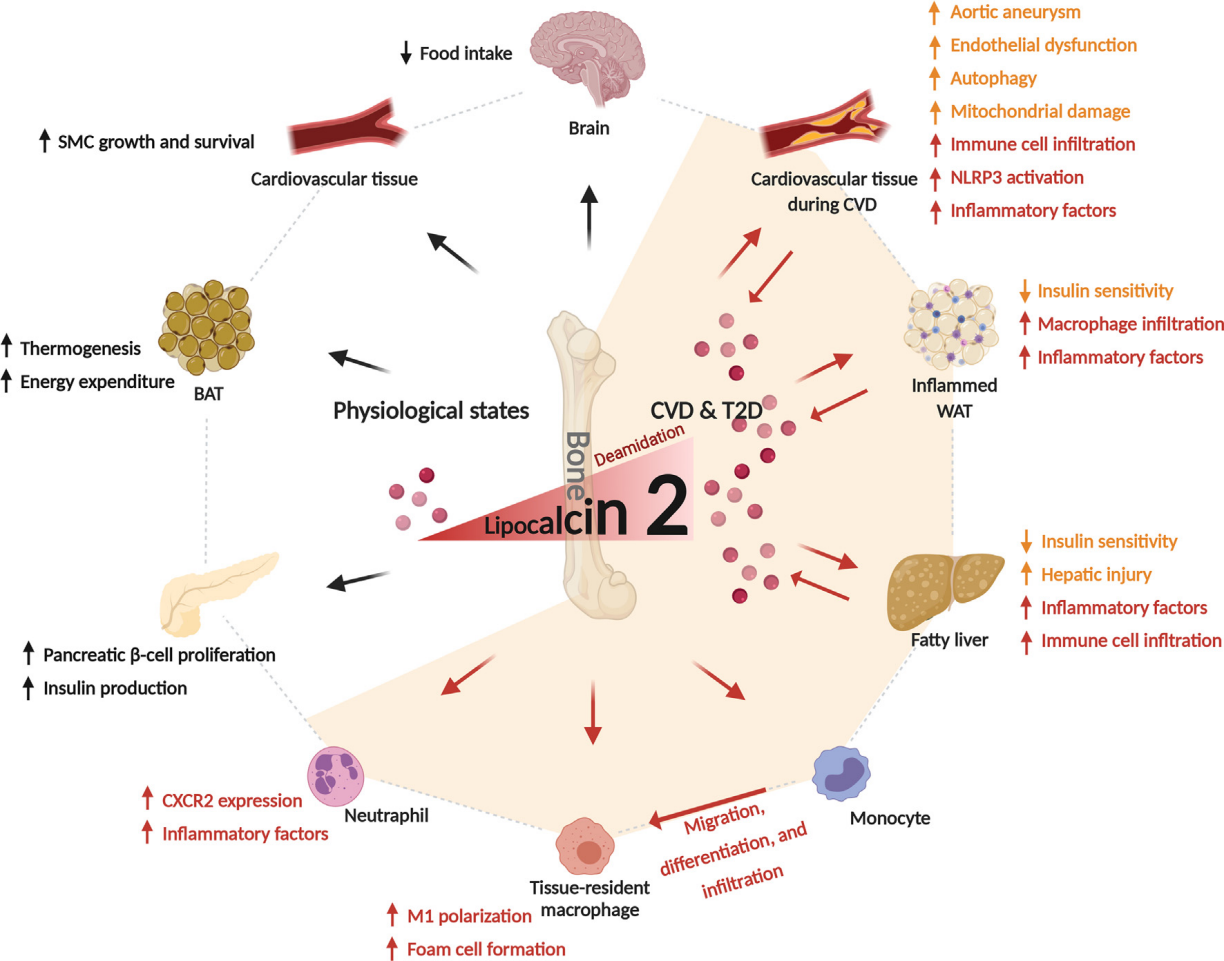
**Roles of lipocalin 2 in cardiometabolic regulation and disease.**Figure 2 incorporates findings from animal models and humans. LCN2 is probably mainly produced in osteoblasts in physiological states [8] and plays an important role in energy homeostasis. In brain, LCN2 controls appetite via its effect on hypothalamus in an MC4R-dependent manner [8]. Furthermore, LCN2 promotes β-cell proliferation and insulin production in pancreas [8]. In addition, LCN2 favors adaptive thermogenesis and energy metabolism in brown adipose tissue [104,111]. In vasculature, LCN2 favors the growth and survival of SMCs [[124], [125], [126]]. In the conditions of T2D and CVD, the circulating levels of LCN2 are considerably increased due to inductive expressions in WAT [[124], [125], [126]], liver [100], and cardiovascular tissues [101], as well as due to enhanced deamidation [95]. Pathological levels of LCN2 potentiates insulin resistance in adipose tissue and liver in T2D [116,117]. During CVD, increased levels of LCN2 contributes to aortic aneurysm [15], endothelial dysfunction [95,123], cell autophagy inhibition [101], and mitochondrial damage in heart tissue [101]. The detrimental roles of LCN2 in cardiometabolic syndromes are closely linked with its pro-inflammatory effects. LCN2 drives immune cell infiltration and NLRP3 inflammasome-induced inflammatory factor expression [100,101]; furthermore, it causes inflammation-related injury in liver and ER stress in smooth muscle cells [100,124]. LCN2 also directly activates immune cells during cardiometabolic syndromes. In monocytes/macrophages, it enhances cell migration, M1 polarization, and foam cell formation [129]. In neutrophils, LCN2 increases CXCR2 expression and the production of pro-inflammatory factors [101]. Short-head arrows: secretion; differentiation. Long-head arrows: suggested LCN2 effects. Orange texts: direct detrimental effects on cardiometabolic tissues in T2D or CVD conditions. Red texts: pro-inflammatory effects in T2D or CVD conditions. LCN2 – lipocalin 2; MC4R – melanocortin-4 receptor; SMCs – smooth muscle cells; T2D – type 2 diabetes; CVD – cardiovascular disease; WAT – white adipose tissue; NLRP3 – nucleotide-binding domain-like receptor protein 3; CXCR2 – the chemokine The meaning was unclear. Thus, changes were made. Please ensure the changes preserve the intended meaning.-X-C motif receptor 2.

Nevertheless, despite the clinical associations and the abovementioned animal studies, numerous mechanistic insights from animal models have demonstrated that LCN2 is important for multiple metabolic processes in various tissues, including appetite control, insulin production, and thermogenesis (Figure 2) [8,104,105,[111], [112], [113]]. The loss of LCN2 in osteoblasts [8] or a global LCN2 deficiency [105] in mice was reported to lead to obesity and insulin resistance because of enhanced food intake. In line with this finding, LCN2 administration in mice resulted in reduced food intake by acting on the melanocortin-4 receptor (MC4R) receptor in the hypothalamus, suggesting an anorexigenic effect for LCN2 [8]. Furthermore, bone-derived LCN2 was shown to exert beneficial effects on the pancreas, because the specific loss of LCN2 from osteoblasts led to impaired insulin secretion and β-cell proliferation [8]. Other studies have indicated that LCN2 may regulate brown adipose tissue (BAT) to enhance thermogenesis and energy expenditure, because LCN2 loss in mice resulted in compromised adaptive thermogenesis, and potentiated diet-induced obesity, dyslipidemia, fatty liver disease, and insulin resistance [[111], [112], [113]]. Consistently, LCN2 overexpression in adipose tissue protected against an aging-induced decline in thermogenic functions and promoted glucose tolerance and lipid homeostasis [104].

The discordant findings from animal models with genetic manipulations are probably due to the genetic and environmental factors selected for these models, leading to differences in LCN2 post-translational modifications including dimeriation and polyamidation [114], and differences in LCN2 production sites. In addition, complex technical issues such as targeting strategies for knockout mice and housing environments could also be responsible for these discrepancies [115].

I*n vitro* evidence also suggests divergent direct metabolic effects of recombinant LCN2 in different cell types. In primary pancreatic islets, the treatment of physiological levels LCN2 (10–100 ng/mL) enhanced glucose-induced insulin secretion [8]. In 3T3-L1 adipocytes, LCN2 treatment (250 ng/mL) increased fatty acid β-oxidation in mature cells [116]. Nevertheless, LCN2 treatment promoted insulin resistance in H4IIe hepatocytes (250 ng/mL) [117] and human adipose tissue (100 ng/mL) [118], and LCN2 knockdown in 3T3-L1 adipocytes increased insulin sensitivity [117]. However, in these studies, either the physiological or pathological levels of LCN2 were used for the treatment; thus, an assessment of whether different levels of LCN2 yield different metabolic outcomes is necessary.

Collectively, although studies have suggested several endocrine functions of LCN2 in energy metabolism, there is little consensus on the subject. To resolve these discrepant findings, further research must identify the modification status and metabolic functions of bone-derived LCN2 versus LCN2 secreted from other tissues and compare their roles in physiological and T2D conditions.

### 2.8 Detrimental role of lipocalin 2 in the development of cardiovascular disease

Observational studies have demonstrated that increased systemic LCN2 levels are associated with the severity of coronary artery disease [119] and increased risk of future CVD events [[120], [121], [122]]. Unlike LCN2 roles in metabolic disorders, mechanistic studies conducted on LCN2-KO mice have demonstrated possible cause–effect relationships between LCN2 and the development of atherosclerotic disease (Figure 2). In Tarín et al., LCN2^−/−^ mice or mice treated with LCN2 antibodies showed a decreased incidence of abdominal aortic aneurysm and reduced aortic expansion compared with that of WT mice or mice treated with IgG (control), respectively [15]. In two other studies, LCN2-deficient mice were protected from dietary challenge-induced endothelial dysfunction, reflected by enhanced blood pressure, impaired endothelium-dependent relaxation, and augmented endothelium-dependent contractions [95,123]. Notably, LCN2 activity seemed to be influenced by its polyamidation status, which contributes to LCN2 removal from general circulation [95]. The administration of the deamidated recombinant LCN2 in mice has led to severe endothelial dysfunction, compared with WT recombinant LCN2 administration [95].

In contrast to these *in vivo* findings, some *in vitro* evidence suggests that physiological levels of LCN2 may have beneficial effects on smooth muscle cells. In a series of studies by Wang and colleagues, the stimulation of 3–30 ng/mL LCN2 in human pulmonary artery smooth muscle cells (HPASMCs) promoted pro-proliferative and anti-apoptotic effects, despite increased ER stress [[124], [125], [126]]. However, it remains possible that LCN2, at much higher pathological levels, leads to cell damage and cell death in the vasculature, but evidence has not been provided.

Collectively, LCN2 enhancement in cardiovascular disorders, especially in the deamidated form, seems to accelerate CVD progression. What remains unclear is whether the changes in LCN2 expression are from physiological sources such as bone, and where the deamidated form of LCN2 is majorly produced. Therefore, during CVD, the modification statuses and roles of the LCN2, which originated from different production sites, must be established and clarified.

### 2.9 Pro-inflammatory role of lipocalin 2 in cardiometabolic disorders

Of all known factors produced by bone, LCN2 is the most closely implicated in inflammation. Notably, LCN2 was initially identified and purified from neutrophil granules [127]. In LCN2 functional studies on innate immune responses, it was suggested to play important roles in bacterial capture [128], and sterile inflammation [27]. However, several later studies, with both *in vivo* and *in vitro* findings, showed that LCN2 provoked inflammatory responses in myeloid cells and cardiovascular tissues during CVD (Figure 2) [15,100,101].

In a mouse study by Tarín et al., LCN2 deficiency or the administration of LCN2 antibodies decreased neutrophil infiltration into the aortic wall during abdominal aortic aneurysm [15]. Other *in vivo* data showed that in low-density lipoprotein receptor-deficient (ldlr ^−/−^) mice fed an HFD and high-cholesterol diet, LCN2 was co-localized with infiltrated macrophages in atherosclerotic plaques [100]. Moreover, in mouse heart tissue, an LCN2 deficiency led to reductions in pressure overload-induced NLRP3 inflammasome activation, accompanied by reduced autophagy and mitochondrial damage, which were reversed by WT LCN2 restoration via recombinant adenovirus expression [101].

*In vitro* findings also suggests a pro-inflammatory role of pathological levels of LCN2. In bone marrow-derived macrophages, LCN2 stimulation (500 ng/mL) up-regulated the M1 macrophage gene markers, *Tnf*, *Nos2*, *Il6*, and *Ccl5* [129]. Furthermore, LCN2 treatment (500 ng/mL) facilitated the formation of macrophage foam cells, via increased lectin-type oxidized LDL receptor 1 (LOX-1) and scavenger receptor A-1 (SRA-1) expression [129]. In monocytic J774A.1 cells, enhanced migration induced by LCN2 treatment (500 ng/mL) was also observed [129]. In another *in vitro* study, LCN2 treatment (200–1000 ng/mL) in human umbilical vein endothelial cells (HUVECs) and HASMCs significantly enhanced secretion of the pro-inflammatory cytokines, IL-8, IL-6, and MCP-1 in a dose-dependent manner [130]. In primary cardiac fibroblasts, LCN2 at 1000 ng/mL enhanced priming and activation of NLRP3-inflammasomes, as evidenced by the increased expression of IL-1β and IL-18, as well as caspase-1 activation [101].

In addition to its inflammatory role in CVD, LCN2 regulates chronic inflammation in other metabolic disorders (Figure 2). In a recent study using mice with diet-induced non-alcoholic steatohepatitis (NASH), the genetic depletion of LCN2 substantially reduced hepatic injury, pro-inflammatory gene expression, and neutrophil and macrophage infiltration [100]. In the same study, LCN2 administration in mice with NASH exacerbated hepatic inflammation and immune cell accumulation in a neutrophil-dependent manner [100]. In another study by Law et al., LCN2 deficiency in mice led to reduced fat mass and less macrophage infiltration in WAT tissue, along with decreased expression of inflammatory gene markers, including *Tnf*, *F4/80*, *Mcp-1*, and *Cd14* [110]. In compatible with the *in vivo* findings, *ex vivo* LCN2 treatment (100–10000 ng/mL) in primary neutrophils enhanced expression of the chemokine C-X-C motif receptor 2 (CXCR2), leading to the activation of extracellular signal-regulated protein kinase 1/2 (ERK1/2), production of pro-inflammatory cytokines, and enhanced cell migration [100].

### 2.10 Sclerostin overview

Sclerostin is a secreted glycoprotein that contains 190 amino acids. Most circulatory sclerostin is produced by osteocytes [131]; however, several other tissue types, such as liver, kidney, and the vascular wall, may also produce glycoprotein [132]. Sclerostin is a strong suppressor of bone mass accrual, because of its antagonizing effects on the Wnt/β-catenin signaling pathway (canonical Wnt pathway) via binding to LDL receptor-related proteins (LRPs) [133]. As such, targeting the paracrine actions of sclerostin in bone is an effective approach for anti-resorptive therapies, such as the administration of the sclerostin monoclonal antibodies romosozumab and blosozumab [134]. Outside the bone, sclerostin is abundantly expressed in the general circulation, with normal physiological levels below 1 ng/mL [135]. Because Wnt/β-catenin signaling pathway is a key regulator of cellular functions, including proliferation, differentiation, and migration in various tissues [136], an endocrine role of bone-derived sclerostin has also been demonstrated [9,137].

### 2.11 Role of sclerostin in promoting adiposity and insulin resistance

It was proposed that the endocrine functions of sclerostin involve negative regulations of glucose and fatty acid metabolism via up-regulating whole-body adiposity, because activators of the canonical Wnt pathway have been implicated as potent inhibitors for adipogenesis [138,139]. Indeed, recent clinical observations have suggested that higher sclerostin levels are associated with higher fat mass, as well as lower peripheral insulin sensitivity [140,141]. Consistent with these clinical reports, a recent mechanistic study demonstrated that sclerostin deficiency in mice resulted in reduced adipose tissue accumulation, improved whole-body and muscle insulin sensitivity and decreased hepatic gluconeogenesis [9]. In another study, the loss of the functional receptor LRP4 in fat tissue led to smaller adipocyte size and increased whole-body insulin response [137]. Additionally, in several mice models with sclerostin overproduction, enhanced fat mass and compromised insulin sensitivity were observed [9,137,142]. However, another recent study reported that sclerostin overexpression may have beneficial effects on energy metabolism by facilitating beige adipogenesis [143], indicating that the physiological roles of sclerostin may be more complex than originally perceived.

In pathological states such as T2D, there are much higher serum sclerostin levels compared with those of healthy controls [144,145], indicating that an increase in sclerostin may contribute to disease development. Consistently, either sclerostin deficiency or the administration of sclerostin antibody protected mice from HFD-induced obesity, hyperglycemia, hyperlipidemia, insulin resistance, and fat deposition in the liver [9]. In *in vitro* adipocytes, pathological levels of sclerostin enhanced cell differentiation, increased fatty acid synthesis, and reduced fatty acid oxidation in either 3T3-L1 (5–20 ng/mL) [146] or primary cells (100 ng/mL) [9,137], contributing to adipocyte hypertrophy.

**Figure 3:**
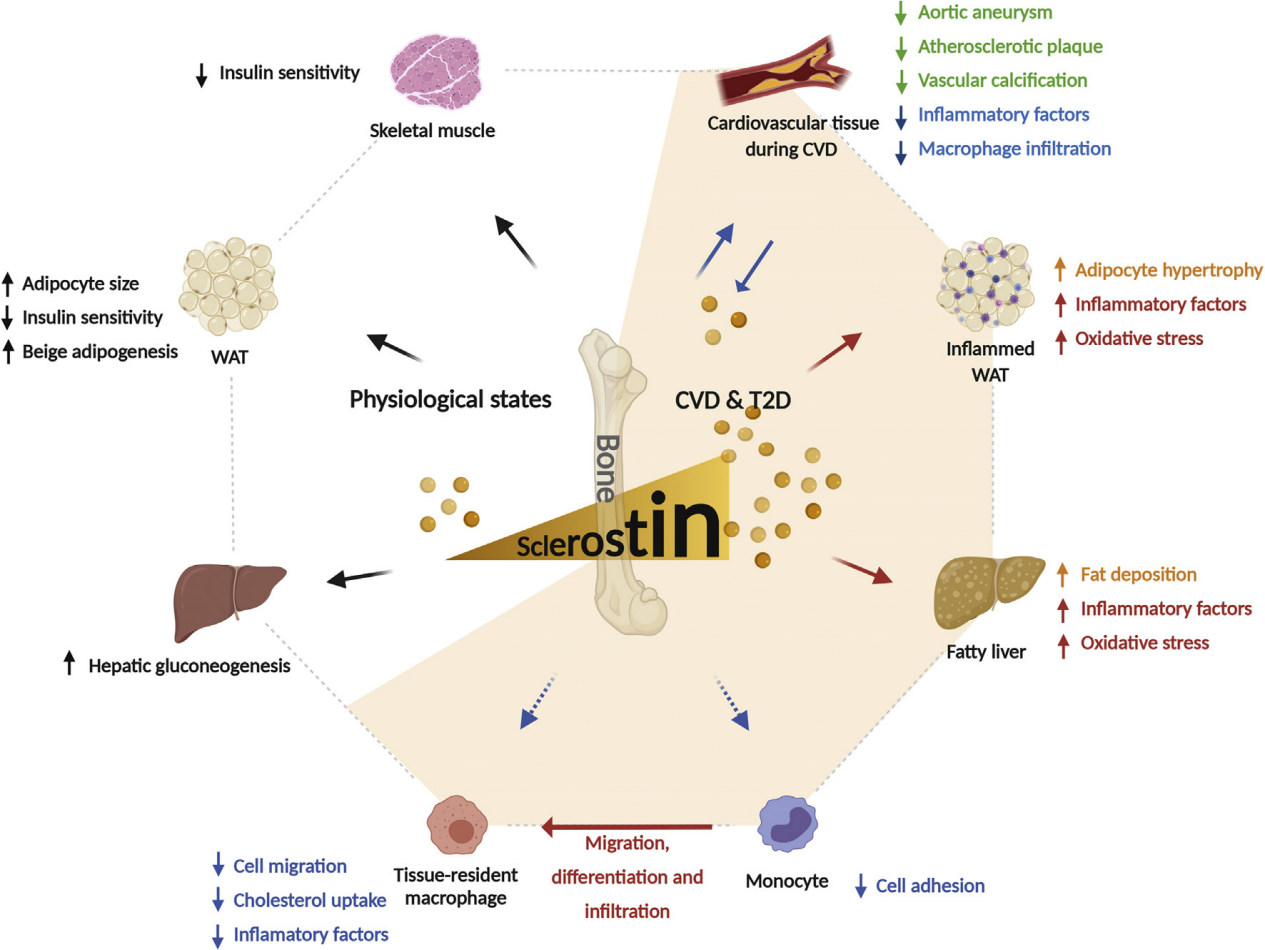
**Roles of sclerostin in cardiometabolic regulation and disease**. Figure incorporates findings from animal models and humans. Sclerostin is predominantly secreted from osteocytes [131], although a smaller amount can also be produced in other tissues, such as vasculature during CVD as a possible compensatory mechanism to counter vascular calcification [132]. In physiological states, sclerostin downregulates energy metabolism via enhancing adiposity and reducing insulin sensitivity [9,137]. It also enhances gluconeogenesis in liver [9]. However, it could also benefit energy expenditure via promoting beige adipogenesis [143]. Sclerostin levels have been reported to elevate in cardiometabolic syndromes [144,145]. Increased sclerostin promotes adipocyte hypertrophy in WAT and fatty liver, leading to an enhancement in inflammatory factor expression and oxidative stress [9,137]. During CVD, sclerostin in vasculature has been suggested to benefit cardiovascular health by reducing aortic aneurysm, decreasing atherosclerotic plaque formation, and preventing vascular calcification [13,150,151]. It also plays an anti-inflammation role by attenuating the inflammatory factor expression and aortic macrophage infiltration [13]. Moreover, sclerostin probably has a direct suppressive effect on monocytes/macrophages by reducing cell adhesion [162,163], inhibiting cell migration [161], decreasing cholesterol uptake [161], and mitigating inflammatory factor production [159,160], via the inhibition of Wnt/β-catenin pathway in these cells. Short-head arrows: secretion; differentiation. Long-head arrows: suggested sclerostin effects. Long-head arrows with dash line: hypothesized sclerostin effects. Green texts: direct beneficial effects on cardiometabolic tissues in T2D or CVD conditions. Blue texts: anti-inflammatory effects in T2D or CVD conditions. Orange texts: direct detrimental effects on cardiometabolic tissues in T2D or CVD conditions. Red texts: pro-inflammatory effects in T2D or CVD conditions. T2D – type 2 diabetes; CVD – cardiovascular disease; WAT – white adipose tissue.

Collectively, despite the majority of studies promoting adverse metabolic effects of sclerostin in physiological and pathological states (Figure 3), its detailed regulatory mechanisms in adipose tissue requires further investigation.

### 2.12 Protective role of sclerostin in vascular calcification

As WNT-β-catenin plays an important role in CVD onset and development [147], potential crosstalk between bone and vasculature via sclerostin has attracted increased attention. Although cross-sectional studies have reported that enhanced sclerostin levels are associated with poor CVD outcomes [148,149], romosozumab (a humanized monoclonal antibody for sclerostin) clinical trials for osteoporosis treatment have suggested an increased CVD risk when compared with that of alendronate [150,151], indicating that sclerostin may benefit cardiovascular health. Recently, sclerostin was proposed to have a preventative action on vascular calcification in complicated atherosclerotic plaques, similar to its role in inhibiting mineralization processes in the bone (Figure 3) [152,153]. In ApoE^−/-^ mice treated with angiotensin II, sclerostin overexpression induced inhibitory effects on the progression of aortic aneurysm and atherosclerosis [13]. Notably, sclerostin expression was considerably increased in different cell types within atherosclerotic plaques [154,155], and it appears to be a counter-regulatory mechanism suppressing vascular calcification development. However, the hormonal action of bone-derived sclerostin in inhibiting vascular calcification requires further investigation. In addition, as sclerostin probably induces hyperglycemia and hyperlipedemia via its actions in adipose tissue, the roles of sclerostin during CVD development may be multifaceted but remain largely unknown. Therefore, further investigations are urgently required.

### 2.13 Complicated role of sclerostin in chronic inflammation regulation

The association of sclerostin with chronic inflammation has been suggested because several inflammatory cytokines stimulate sclerostin expression in osteocytes under pathological conditions, including obesity, diabetes, and CVD [[156], [157], [158]]. However, whether sclerostin modulates chronic inflammation during cardiometabolic diseases remains poorly understood and controversial according to findings of recent studies. In HFD-fed *Sost*^−/−^ mice, the expression levels of the inflammatory markers *Ccl2*, *Ccl3*, and *Ccl4* and the oxidative stress genes *Nox4*, *Sod1*, and *Sod2* were decreased in WAT and liver when compared with WT controls. This finding was consistent with reduced adiposity in these mice [9]. By contrast, the global overexpression of *Sost* in ApoE^−/−^ mice was shown to reduce circulating levels of pro-inflammatory factors and aortic macrophage infiltration during aortic aneurysm and atherosclerosis, induced by angiotensin II infusion [13].

Understanding the direct effects of sclerostin on monocytes/macrophages would greatly enhance the characterization of its role in chronic inflammation. Despite the insufficient evidence on the direct effects of sclerostin in myeloid cells, many studies have demonstrated a suppressive effect of sclerostin on monocytes/macrophages inflammation and migratory capacity via the Wnt/β-catenin signaling pathway [[159], [160], [161], [162], [163]]. In support of this notion, in both Thp-1 and human primary monocytes, the activation of the Wnt/β-catenin pathway by lithium or Wnt3a increases monocyte adhesion to endothelial cells [162,163]. In human macrophages, Wnt signaling reduction via LRP-5 deficiency resulted in decreased cholesterol uptake and cell migration [161]. Furthermore, lipopolysaccharides (LPS)-induced β-catenin accumulation has been demonstrated to be involved in cell migration in RAW264.7 macrophages, and inhibition of the Wnt/β-catenin pathway via a small-molecule inhibitor attenuated the expression of inflammatory factors [159,160].

In summary, the evidence points toward an inhibitory effect of sclerostin on monocyte/macrophage inflammation via the Wnt/β-catenin pathway. Because sclerostin promotes adiposity—in turn—initiates inflammatory responses, the role of sclerostin in chronic inflammation is probably complicated and may vary by pathological condition (Figure 3). Therefore, further research, particularly on the direct effects of sclerostin on inflammatory pathways in adipocytes and monocytes/macrophages, is required before decisive conclusions can be reached regarding its role in chronic inflammation.

## 3 Conclusions and perspectives

The endocrine action of bone-derived hormones is an emerging, topical branch of endocrinology and pathophysiology research. In physiological states, ucOC, LCN2, and sclerostin exert hormonal regulations in energy metabolism and cardiovascular health. More importantly, accumulating evidence suggests that the perturbance in the production and modification of these hormones is closely linked to the development of T2D and CVD, via regulating several causal factors, which probably includes chronic inflammation. However, the findings that suggest the roles of bone-derived hormones in chronic inflammation are scarce compared with the findings of other well experimentally supported areas in bone endocrinology. Thus, a considerable amount of further evidence to support this hormonal function of bone remains urgently needed from animal and human studies.

Overall, evidence from the literature supports an anti-inflammatory role of ucOC in combating T2D and CVD, aligned with its beneficial metabolic and cardiovascular effects, possibly via the suppression of ER stress and oxidative stress. By contrast, enhanced circulating levels and a deamidated form of LCN2 in pathological states appear to deteriorate CVD conditions, partly via inflammation exacerbation, although physiologically, it benefits energy metabolism mainly via anorexigenic effects and enhancing thermogenesis in BAT. As to sclerostin, the enhancement of sclerostin levels probably perturbs glucose and lipid homeostasis by promoting adiposity during the development of T2D. However, sclerostin expressed in cardiovascular tissues may play a role in inhibiting atherosclerosis progression by reducing vascular calcification. The role of sclerostin in chronic inflammation remains complicated, promoting inflammatory reactions in WAT via enhancing adiposity despite an implicative anti-inflammation role in vasculature and monocytes/macrophages.

Based on this accumulated knowledge, ucOC administration seems to have the most therapeutic potential for the management of T2D, CVD, and chronic inflammation, and the antagonization of LCN2 and sclerostin during cardiometabolic disease may also yield beneficial effects. However, the following questions must be answered in further mechanistic and pre-clinical research: (1) Compared with non-bone-produced proteins, especially LCN2, do bone-derived factors play major roles in pathological conditions? (2) Do these factors exert direct effects on monocytes/macrophages, especially ucOC and sclerostin? (3) Will combination treatment involving the modulation of circulating levels of several bone-derived hormones result in improved therapeutic outcomes in treating T2D, CVD, and chronic inflammation? Finding answers to these questions will help elucidate the complex mechanisms underlying the crosstalk between the bone and metabolic and cardiovascular tissues. In the context of drug discovery, a comprehensive understanding of these mechanisms would be a substantial step toward discovering novel treatments, targeting bone-derived hormonal pathways, in combating cardiometabolic disorders.

## Ethics approval and consent to participate

Not applicable.

## Consent for publication

Not applicable.

## Availability of data and materials

Not applicable.

## Funding

This work was supported by the 10.13039/501100000925National Health and Medical Research Council (NHMRC) of Australia in the form of a project grants 1156634 & 1158242 to K.L. The salary of J.L. is supported by a 10.13039/501100001030National Heart Foundation Future Leader Fellowship (ID: 102817).

## Authors' contributions

XL and KL were responsible for the general conception and design of the review paper. All authors contributed in writing the manuscript. All authors approved the final manuscript.
